# Exercise-Induced Muscle Damage and Oxidative Stress

**DOI:** 10.3390/antiox7040050

**Published:** 2018-03-28

**Authors:** Athanasios Z. Jamurtas

**Affiliations:** Department of Physical Education & Sport Science, University of Thessaly, Karies, Trikala 42100, Greece; ajamurt@pe.uth.gr; Tel.: +30-243-1047-054; Fax: +30-243-1047-054

Exercise-induced muscle damage (EIMD) is associated with muscle soreness or discomfort and a marked decline of muscle strength during the first 12–72 hours post-exercise [1]. Furthermore, EIMD leads to the onset of an inflammatory response that is associated with the activation of leukocytes, muscle oedema, deterioration of muscle function, delayed-onset of muscle soreness (DOMS), and several intracellular events that aim to restore the integrity and function of the affected muscle [2]. Oxidative stress, on the other hand, indicates a condition where the cellular production of pro-oxidant molecules exceeds the ability of the antioxidant system to reduce reactive oxygen or nitrogen species (RONS). Research indicates that oxidative stress is evident following muscle damaging exercise [3]. Perturbations in oxidative stress seem to have a potent role in the adaptation process following EIMD. The purpose of this Special Issue of *Antioxidants* was to highlight recent developments on the field of EIMD and its association with inflammation and oxidative stress. Three review papers and five articles with original data were published. 

The review paper by Gomez-Cabrera reviewed studies that examined the treatment with cyclooxygenase-inhibiting drugs and how these can modulate responses to one bout of resistance exercise [4]. Their paper highlights three main points: (1) COX-inhibiting drugs modulate the protein synthesis response to resistance exercise and to training differently; (2) contrary to what has been reported with antioxidants, chronic consumption of COX inhibitors during exercise training does not appear to interfere with muscle mass and strength gains expected from resistance-exercise-training regimens; and (3) recent studies open up the possibility of treating sarcopenia with COX-inhibiting drugs. The second review paper by Avloniti et al. discussed an important and less examined issue that deals with exercise-induced oxidative stress responses in the paediatric population [5]. That review mentions that acute exercise induces age-specific transient alterations in both oxidant and antioxidant markers in children and adolescents. However, these responses seem to be affected by factors such as the training phase, training load, fitness level, mode of exercise, etc. In relation to chronic adaptation, training seems to positively modulate the antioxidant system in adolescents to a similar degree as it does with adults. Furthermore, the possible role that oxidative stress might play in growth is discussed, as is the role obesity plays on the antioxidant responses in the peripubertal period. The third review published in this special issue was by Webb et al. [6] and the consequences of the exercise-induced oxidative stress on cellular signalling processes are discussed. An attempt was made by the authors to elucidate mechanisms by which exercise-induced oxidative stress can result in beneficial responses locally in muscle. That happens because RONS can work not only as local autocrine signalling molecules, but as paracrine/endocrine molecules, as well affecting several other tissues, and the body as a whole. 

In the paper by Burns et al., chronic tempol, a membrane permeable superoxide dismutase mimetic, supplementation was examined in muscular dystrophy (*mdx*)-afflicted mice [7]. Tempol supplementation was found to recover the force-generating capacity and metabolic enzyme activity in a *mdx*-affected diaphragm, suggesting that such molecules could play a role in finding therapeutic strategies to treat neuromuscular diseases. An interesting paper by Eccardt et al. highlighted the importance of trans plasma membrane electron transport (tPMET) and ascorbate efflux by skeletal muscle in the regulation of muscle cell integrity and avoidance of metabolic dysfunction [8]. Using C2C12 myotubes, the authors showed that muscle cells are capable of tPMET and ascorbate efflux and that this function is supported by GLUT-1, but not GLUT-4 or insulin, highlighting the important role GLUT-1 plays in regulating RONS levels in muscle cells. Hsu et al. evaluated the dose response effects of ginseng steroids on redox status under resting and exercised conditions [9]. They utilized the rat model and supplemented the animals with low, medium, and high doses (20, 40, and 120 mg per kg) of ginseng steroids. Their results showed that, under resting conditions, supplementation with a high dose of ginseng steroids might lead to increased lipid peroxidation and a lower ratio of GSH/GSSG, whereas low and medium doses can result in higher activities of selected antioxidant enzymes. Furthermore, low and medium doses of ginseng steroids provided better protection against exercise-induced lipid peroxidation, whereas the high dose had the opposite effect, suggesting that caution should be taken when supplementation with high ginseng steroids is to be followed. McLeay et al. examined the effects of taurine supplementation on performance and muscle damage following eccentric exercise [10]. Results from this study indicate that taurine supplementation, starting immediately after an intense eccentric exercise bout, results in an attenuation of maximal eccentric torque decrements without, however, affecting the creatine kinase (CK) responses. 

Finally, Oosthuyse, and Bosch, in a study where they examined the serum CK and DOMS following a downhill exercise bout in men and women, found that CK was restored back to pre-exercise levels quicker compared to men, whereas DOMS did not follow the same pattern [11]. Men’s DOMS was restored to initial levels within 48 h after the exercise bout, whereas women still had elevated muscle soreness feelings even 72 h post-exercise. Furthermore, results from this study indicate that menstrual cycle phase plays a role on the subjective feeling of pain following eccentric exercise indicating that oestrogens might play a role on this phenomenon.

There are certainly many more things to learn about the interplay between molecules released when a muscle is injured and the perturbations of the redox system. Elucidating such mechanisms will result in optimal treatments of the injured muscle and prompt athletic recovery. 

## References

[1] Jamurtas A.Z., Theocharis V., Tofas T., Tsiokanos A., Yfanti C., Paschalis V., Koutedakis Y., Nosaka K. (2005). Comparison between leg and arm eccentric exercise of the same relative intensity on indices of muscle damage. Eur. J. Appl. Physiol..

[2] Fatouros I.G., Jamurtas A.Z. (2016). Insights into the molecular etiology of exercise-induced inflammation: Opportunities for optimizing performance. J. Inflamm. Res..

[3] Paschalis V., Nikolaidis M.G., Fatouros I.G., Giakas G., Koutedakis Y., Karatzaferi C., Kouretas D., Jamurtas A.Z. (2007). Uniform and prolonged changes in blood oxidative stress after muscle-damaging exercise. In Vivo.

[4] Gomez-Cabrera M., Viña J., Ji L. (2016). Role of Redox Signaling and Inflammation in Skeletal Muscle Adaptations to Training. Antioxidants.

[5] Avloniti A., Chatzinikolaou A., Deli C., Vlachopoulos D., Gracia-Marco L., Leontsini D., Draganidis D., Jamurtas A., Mastorakos G., Fatouros I. (2017). Exercise-Induced Oxidative Stress Responses in the Pediatric Population. Antioxidants.

[6] Webb R., Hughes M., Thomas A., Morris K. (2017). The Ability of Exercise-Associated Oxidative Stress to Trigger Redox-Sensitive Signalling Responses. Antioxidants.

[7] Burns D., Ali I., Rieux C., Healy J., Jasionek G., O’Halloran K. (2017). Tempol Supplementation Restores Diaphragm Force and Metabolic Enzyme Activities in mdx Mice. Antioxidants.

[8] Eccardt A., Bell T., Mattathil L., Prasad R., Kelly S., Fisher J. (2017). Trans-Plasma Membrane Electron Transport and Ascorbate Efflux by Skeletal Muscle. Antioxidants.

[9] Hsu M., Yu S., Korivi M., Jean W., Lee S., Huang C., Liao Y., Lu J., Kuo C. (2017). Hormetic Property of Ginseng Steroids on Anti-Oxidant Status against Exercise Challenge in Rat Skeletal Muscle. Antioxidants.

[10] McLeay Y., Stannard S., Barnes M. (2017). The Effect of Taurine on the Recovery from Eccentric Exercise-Induced Muscle Damage in Males. Antioxidants.

[11] Oosthuyse T., Bosch A. (2017). The Effect of Gender and Menstrual Phase on Serum Creatine Kinase Activity and Muscle Soreness Following Downhill Running. Antioxidants.

